# A mixed methods evaluation of an antimicrobial prescribing clinical decision support system app

**DOI:** 10.1038/s44259-025-00146-8

**Published:** 2025-08-18

**Authors:** William J. Waldock, Mark Gilchrist, Hutan Ashrafian, Ara Darzi, Bryony Dean Franklin

**Affiliations:** 1 Institute of Global Health Innovation, Guild, Imperial College London, London, UK; 2 Imperial College Healthcare NHS Trust, London, UK; 3 NIHR Northwest London Patient Safety Research Collaboration, London, UK; 4 UCL School of Pharmacy, London, UK

**Keywords:** Computational biology and bioinformatics, Biomarkers, Health care, Medical research

## Abstract

This study evaluated how the usability and accessibility of a digital antimicrobial prescribing app influences clinical decision-making. Using a convergent parallel mixed methods design, the study assessed app usage patterns with surveys and interviews to identify common barriers. Among 700 users at a tertiary hospital, 61 completed the survey (7.3% response rate), including 52 prescribers. Additionally, 20 prescribers participated in interviews. While 87% found the guidelines relevant, only 52% rated navigation as easy, and 34% reported slower decision-making compared to other clinical decision support systems (CDSS). App use peaked during morning rounds (8–11 AM). Key challenges included navigation inefficiencies (59%), technical barriers, limited onboarding, and concerns around clinical AI transparency. Interviews highlighted frustration with excessive steps and a desire for simpler guideline access. Findings highlight the need for user-friendly CDSS tools integrated into clinical workflows, and stress the importance of stakeholder co-design to improve medication safety.

## Introduction

Digital health technologies, including clinical decision support, have potential to improve antimicrobial prescribing^1^. The misuse of antibiotics exacerbates resistance^2^, leading to prolonged illnesses, higher healthcare costs, and increased mortality^3^. Although guidelines are widely available, accessing and applying them in a clinical setting remains challenging. Major barriers to adopting clinical decision support systems include lack of clinician trust, poor usability, and misalignment with real-world decision-making processes^4^. Prescribers often face obstacles such as unclear navigation within existing CDSS apps, a lack of mobile accessibility, and limited integration into clinical workflows^5^. Eolas Medical is a digital knowledge management CDSS *(clinical decision support system)* app widely used across NHS trusts in England to store and present local clinical guidelines, including those for antimicrobial prescribing. Unlike other CDSS apps, it does not currently provide algorithmic calculations, such as sepsis scoring or pneumonia severity indices. Its primary function is to surface hospital-approved guidance in an accessible format via mobile or desktop.

Antimicrobial guidelines play a crucial role in managing infections in healthcare settings. However, the use and adherence^6^ to these guidelines can vary, leading to potential suboptimal outcomes in patient care. A systematic review of clinical decision support systems (CDSS) for antimicrobial management in respiratory infections found a pattern of failure to capture real-world decision-making complexity and an underestimation of behavioural influences^7^. Rawson et al. have expertly identified that there are key decision points where interventions can be most effective^8^ but diagnostic results are not actioned in a standardised way^9^. Inaccessible, confusing, and hard to follow clinical guidelines have been found to contribute to prescribing and medication administration incidents^10^, often due to difficulties in using the information offered, including antimicrobial prescribing.

Digital prescribing support CDSS apps seek to streamline access to these clinical guidelines in response to diagnosis. Despite the global deployment of CDSS apps, there is limited evidence on their real-world usability and accessibility in presenting antimicrobial prescribing guidelines. This study addresses this critical gap. This project aims to evaluate the current utility of antimicrobial guidelines accessed through a prescribing support app at a London teaching hospital organisation with three sites and gather structured feedback from healthcare professionals.

This evaluation aims to provide actionable insights that will enhance clinical decision-making about antimicrobial prescribing. The study objectives were therefore threefold^1^: to evaluate the usability, accessibility, and relevance of the antimicrobial guidelines as presented through a CDSS app^2^, to identify barriers commonly faced by healthcare professionals in using the app to support safe antimicrobial prescribing, and^3^ to gather structured feedback to inform future CDSS app development.

## Results

### Survey response rates

Of 700 app users at the NHS hospital trust, 61 respondents (response rate: 7.3%) completed the survey, comprising 52 prescribers (85%) and 9 non-prescribers (15%). The analysis of user feedback from both survey and interviews reveals several key insights (Table 1) regarding the app’s presentation of prescribing guidelines and user experience. Qualitative thematic coding (Supplementary Table 1) further elaborated on these findings. Many users requested simplified navigation pathways and features such as a “favourites” option for quicker access to frequently used guidelines. MicroGuide was a previous app used to support antimicrobial prescribing decisions prior to July 2024. Comparisons with MicroGuide revealed resistance to transitioning to the Eolas app, largely due to perceived inefficiencies and limited onboarding support. Both survey and interview datasets indicate that navigation, decision efficiency, and content clarity should be the primary focus to enhance usability.

**Table 1:** Survey Results Overview

Category	Description	Details
Participant Demographics	Number of Participants	61 participants
	Prescribers vs Non-Prescribers	52 prescribers (85%) and 9 non-prescribers (15%)
	Roles	26% medical consultants, 15% pharmacists, 59% resident doctors
Usage Patterns	Reported frequency of Use	28% daily, 37% weekly, 6% monthly, 8% less than monthly, 8% never
	Most Common Use Times	48% (8–11 AM), 33% (11 AM–4 PM), smaller groups at 1 AM–8 AM and 5 PM–8 PM
	Primary Use Case	90% access antimicrobial prescribing guidelines
User Feedback (Likert Scales)	Ease of Navigation	52% rated 4 (easy)
	Relevance of Information	87% agreed/strongly agreed (53% agree, 34% strongly agree)
	Efficiency	59% efficient, 34% slower than comparable tools
Feature Evaluation	Most-Used Features	Antimicrobial guidelines, drug interactions, penicillin allergies, surgical prophylaxis, Respiratory Tracts Infections, Gastrointestinal infections, meningitis, sepsis treatment, body systems
	Redundant Elements	Excessive links to National Institute for Health and Care Excellence and the British National Formulary, duplicate guideline options, unclear navigation to organization page, non-defaulting to organisational homepage

### Usability and navigation

Survey results highlighted that users felt navigation steps on the app were excessive steps and had difficulty locating local protocols, although the search function was generally found helpful. Interview feedback portrayed navigation as cumbersome, slow, and unintuitive, with many users expressing frustration over the excessive number of clicks needed to locate information. Comparisons with MicroGuide further underscored design complexity of Eolas as a major usability barrier, with repeated concerns about difficulties in accessing local guidelines. While a minority of users found the navigation effective, the majority suggested streamlined navigation, reduce clicks, and improve the interface’s intuitiveness.

### Antimicrobial guidance and content gaps

Interviewees identified antimicrobial guidance as the most frequently used feature but raised concerns about content gaps and formatting inconsistencies. Users requested that antimicrobial guidance be made more prominent within the main menu and suggested adding quick-reference CDSS apps for easier access. Both survey and interview data highlighted the app’s strength in antimicrobial prescribing but emphasized the need for improved content organization and integration. There were consistent calls for better formatting, the removal of duplicate information, and closer alignment with national prescribing standards such as NICE and UKHSA.

### Comparative user experience: eolas vs. microGuide

MicroGuide was the antimicrobial guidelines app previously used at the study site until July 2024. Its simpler interface and navigation were cited by users as preferable in many cases. According to both survey and interview data, using Eolas was perceived to be more complex than MicroGuide, often citing the additional navigation steps required. While some users appreciated Eolas’ intuitive design elements, functionality was consistently prioritized over aesthetics by participants. Interview feedback reinforced survey findings, frequently describing MicroGuide as more efficient and user-friendly. Aesthetic improvements in Eolas were seen by participants as secondary to usability, with recurring recommendations to emulate MicroGuide’s streamlined interface. The overall preference for simplicity and efficiency supported suggestions to reduce navigation steps and adopt familiar design patterns.

### Clinical decision support and content deficiencies

Survey responses revealed significant concerns about incomplete dosing guidance and a strong preference for scenario-based prescribing CDSS apps. Another key gap was lack of comprehensive advice for patients with penicillin allergies across multiple clinical syndromes. According to specialist infectious disease registrars who participated, in contrast to other trusts offering detailed guidance, the host hospital guidance presented on Eolas often defaults to advising a call to infection teams, leading to delays and added specialist workload. Structural inconsistencies within the app further exacerbated usability issues, with features such as the Ear, Nose and Throat section being incorrectly categorized under respiratory conditions, complicating access to relevant information. Interviewees also stressed the need for expanded dosing guidance, and improved hyperlink integration.

### Workflow inefficiencies and user recommendations

Workflow inefficiencies were a prominent concern, with users frequently citing excessive clicks and complex processes as significant obstacles. Many expressed a desire for bookmarking features to quickly access frequently used guidelines. Interviewees’ recommendations focused on introducing bookmarking, simplifying workflows, and reducing the number of steps needed to complete tasks. While users acknowledged the potential for machine learning in antibiotic prescribing, they emphasised the importance of simplicity and transparency to avoid disrupting workflows. Interviewees stressed that clinician trust depends on transparent systems, warning that complex or opaque features would likely face scepticism. Consequently, any machine learning apps should be introduced cautiously, prioritising usability and ensuring that new features enhance rather than complicate clinical workflows.

## Discussion

Of 700 app users at the NHS hospital trust, 52 prescribers (85%) and 9 non-prescribers (15%) (response rate: 7.3%) completed the survey. Additionally, 20 prescribers participated in semi-structured interviews. Survey data revealed that 87% of respondents found the antimicrobial prescribing guidelines relevant, while 52% rated the app’s navigation as ‘easy’. 34% reported slower decision-making compared to alternative apps. Usage patterns indicated peak activity between 8 and 11 AM (48%), aligning with clinical rounds and surgical lists. The analysis suggests critical gaps in current clinical decision support apps that hinder efficient patient management and increase specialist workload.

The findings suggest that the app’s navigation system requires significant refinement to meet user expectations. Streamlining navigation, reducing the number of clicks, and creating a more intuitive interface are critical to improving overall user satisfaction. Antimicrobial guidance remains a core strength of the app but requires better content organisation and visibility. Making this feature more prominent within the main menu and introducing quick-reference apps could significantly enhance usability. Addressing formatting inconsistencies and aligning content with national prescribing standards would further improve user trust and efficiency.

The gaps identified in clinical decision support apps, particularly around scenario-based prescribing and risk stratification, present opportunities for improvement. Introducing targeted prompts and clearer protocols for penicillin allergy would be beneficial. Expanding guidance for patients with penicillin allergies would also empower clinicians to make safer, independent prescribing decisions, reducing reliance on microbiology. The comparison with previous services at the hospital highlights a clear user preference for simplicity and efficiency over aesthetic enhancements. While some users recognized Eolas’ design strengths, the majority valued streamlined functionality. Emulating MicroGuide’s efficient navigation and interface simplicity would align Eolas with user expectations and improve its overall reception.

Workflow inefficiencies remain a significant barrier to user satisfaction. Introducing features such as bookmarking and simplifying multi-step processes would address common frustrations for users. While users acknowledged the potential for machine learning in improving prescribing practices, they stressed that such features must be transparent, easy to use, and carefully piloted to avoid disrupting workflows. Maintaining a balance between innovation and usability is essential to gaining clinician trust and ensuring widespread adoption.

The use of SUPR-Q facilitated the assessment of usability, trust, and user loyalty, making it particularly relevant for a clinical decision support app such as Eolas Medical, where trust in the data presented is critical. The findings indicate that while users recognize the value of the app’s antimicrobial guidance, improvements in content organization and visibility are necessary to strengthen trust—key aspects evaluated through SUPR-Q. Addressing formatting inconsistencies and aligning content with national prescribing standards would further enhance confidence in the app’s reliability. Meanwhile, SUS provided a rapid and reliable metric for overall usability, making it ideal for benchmarking the app against another. The evaluation design incorporated a combination of interview discussions and surveys, informed by principles from SUPR-Q, QUIS, and additional frameworks such as the Software Usability Measurement Inventory (SUMI)^11^ and NASA Task Load Index (NASA-TLX)^12^. QUIS allowed for a detailed examination of user satisfaction with specific aspects of the app’s interface and functionality. The findings highlight that the navigation system requires significant refinement, including streamlining pathways, reducing clicks, and creating a more intuitive interface. These insights, derived from QUIS, inform actionable design improvements, such as introducing quick-reference apps and repositioning core features like antimicrobial guidance for better accessibility. The SUS-based usability assessment revealed workflow inefficiencies as a significant barrier, emphasizing the need for features like bookmarking and simplified multi-step processes to reduce frustration. SUMI contributed insights into user satisfaction, revealing that while some users appreciated Eolas’ design strengths, most prioritized streamlined functionality. NASA-TLX captured data on cognitive load, highlighting that excessive navigation complexity increases task burden, particularly in high-pressure clinical settings. This supports the recommendation to introduce targeted prompts and clearer protocols for scenario-based prescribing and risk stratification. Expanding guidance for penicillin-allergic patients would empower clinicians to make safer, independent prescribing decisions, reducing reliance on microbiology input. This multi-faceted evaluation approach ensured that usability barriers were not only identified but also assessed for their broader implications on clinical workflows and patient care. While users acknowledged the potential for machine learning in enhancing prescribing practices, they stressed the importance of maintaining transparency and usability, reinforcing the need for a balanced approach to innovation.

This evaluation of the Eolas Medical app can be synthesised with existing peer-reviewed literature to inform the next generation of development, enhancing patient safety standards in five ways. This evaluation synthesised key themes from the thematic analysis and quantitate results into the novel SMART framework (Stakeholder engagement, Medication safety, Accessibility, Research on long-term effectiveness, and Transparent machine learning), developed as a direct output of this study and to provide a structured approach to improving clinical decision support apps whilst learning from similar studies.

### Stakeholder engagement and implementation strategies

Clinician and patient involvement are critical drivers of successful and sustained organisational adoption of clinical decision support systems (CDSS). Consistent with challenges reported across similar digital health interventions, our evaluation highlights usability barriers, including excessive navigation steps and difficulty locating local protocols, which can impede routine use. Organisational adoption of CDSS apps requires more than technical deployment; as recent literature underscores, it depends on aligning the tool with clinical workflows, fostering a culture of co-production, and ensuring visible support from leadership^1,4^. Systematic reviews of antimicrobial prescribing CDSS tools emphasise that early and continuous clinician engagement, iterative usability testing, and real-time feedback mechanisms are essential for maximising both adoption and perceived utility^1,13^. For Eolas Medical, embedding iterative usability evaluation beyond initial survey responses, engaging prescribers in content curation, and establishing structured feedback loops would support integration into routine practice. While app-based platforms such as Eolas offer significant advantages in accessibility and rapid dissemination of up-to-date guidance compared to traditional paper-based resources, the broader evidence regarding their impact on prescribing behaviours and clinical outcomes remains limited. Future implementation strategies should therefore prioritise user-centred design, workflow integration, and robust evaluation of clinical effectiveness alongside usability and adoption metrics.

### Medication safety

Behavioural science has a significant impact on medication safety in general, particularly in optimising general prescribing practices and improving adherence to guidelines across diverse specialties^14,15^. Behavioural domains have been found to be essential in understanding Parkinson’s disease prescribing^16^. The implementation of decision support alerts and structured dosing guidance has been effective in reducing prescribing errors^17,18^, alongside enhancing antimicrobial stewardship^19^. Moreover, related literature described an apixaban dosing CDSS that ensured comparable appropriateness to manual order approvals^20^, primary care CDSS influenced prescribing towards guideline adherence^21^ and an antibiotic prescribing CDSS improved empirical treatment selection but required more outcome-focused research^13^. Therefore, Eolas development should include enhanced antimicrobial prescribing guidance to ensure complete dosing information and better hyperlink integration, introduction of structured dosing apps for paediatrics and surgical settings, as seen in successful CDSS implementations.

### Accessibility and workflow integration

Efforts to implement CDSS underscore the necessity of seamless workflow integration to maximise clinician adoption. Related literature indicates that poorly designed interfaces and excessive alerts can lead to alert fatigue and hinder adoption; a community pharmacy CDSS improved medication safety but required better integration for usability^22^. Meanwhile, EHR-based CDSS alerts successfully modified prescribing behaviours when minimally disruptive^23^, and primary care prescribing apps are trusted but usability enhancements were still needed^24^. Improvements to Eolas could involve improving navigation efficiency to reduce excessive clicks, implementing advanced search functionality and direct access to guidelines, and enabling bookmarking and favouriting of frequently used features to streamline decision-making.

### Research on long-term effectiveness

A recurring limitation across CDSS literature is the lack of long-term evaluation of patient outcomes. Most research has focused on prescribing behaviour rather than clinical impact on morbidity and mortality. A systematic review has highlighted the efficacy of CDSS in improving prescribing, but outcome-based research is scarce^25^. Few studies assess long-term sustainability, making it difficult to determine the enduring impact on patient care. Therefore, Eolas can be improved by incorporating continuous feedback loops within the app (e.g., user surveys, analytics) to measure engagement over time, aligning with national frameworks such as NICE guidelines and local resistance data to support long-term usability, whilst also conducting follow-up studies on whether Eolas improves patient safety and antimicrobial stewardship.

### Transparent machine learning

Our evaluation highlighted participants’ concern that improvements to Eolas be understandable to users and prescribing advice trustworthy. The role of AI-driven clinical decision support in prescribing has been highlighted in recent CDSS evaluations but concerns about alert fatigue and transparency remain significant^26,27^. Automated alerts and AI-based recommendations have been found to be effective when relevant and actionable^28^, whilst the overuse of AI-driven alerts led to clinician frustration and reduced adherence to guidance^29^. Most significantly, transparency in AI decision-making is crucial for clinician trust^30–32^. This principle for fair interpretable machine learning has been expertly deployed by Bolton et al. for personalising the intravenous to oral antibiotic switch decision making^33^. Eolas may be enhanced through implementing stochastic models cautiously, ensuring transparency and clear explanations for AI-driven suggestions to increase clinician confidence, learning from alert fatigue studies, and ensuring AI interventions remain clinically useful rather than intrusive.

The evaluation faced notable limitations. The low survey response rate (7.3%) introduces potential non-response bias. It is possible that respondents were disproportionately users with strong opinions; either highly critical or particularly engaged with the app. To mitigate this, we triangulated findings with interview data (purposively sampled for diversity) and usage logs to ensure broader context and validate emergent themes. The generalisability of the results was constrained by the study’s focus on a single healthcare setting. This limitation restricted the applicability of the findings to other healthcare systems with differing operational contexts and technological infrastructures. Furthermore, the qualitative data derived from interviews was vulnerable to inherent biases, including the overrepresentation of polarized viewpoints. Notably, as quantitative usage data for MicroGuide from the prior implementation are unavailable, the comparison remains qualitative. Participants may have felt compelled to express either overly positive or overly critical opinions, influenced by their colleagues when more than one participant was on the call. These limitations influenced the design of the evaluation framework and its interpretation. Efforts were made to mitigate biases through triangulation, combining data from surveys, interviews, and interaction logs to ensure a more balanced understanding. To address the generalisability challenge, further work can conduct future studies across diverse healthcare systems, as also corroborated by Kilbourne et al.^34^ and Moullin et al.^35^.

This service evaluation assessed the usability, accessibility, and relevance of microbiology guidelines delivered through an antimicrobial prescribing app. The evaluation highlighted critical areas for improvement, particularly in navigation, content integration, and workflow efficiency. While the app demonstrated strengths in antimicrobial guidance and prescribing support, usability challenges limited effectiveness. Addressing these challenges through streamlined navigation, enhanced content organization, and cautious implementation of advanced features such as machine learning will be key to improving user experience and clinical utility. Future work should include evaluations in diverse healthcare settings and the iterative refinement of the app based on continued user feedback, ensuring sustained impact in clinical practice. Future work should also incorporate structured stakeholder engagement and iterative usability testing at implementation, ensuring new CDSS apps meet clinician needs while supporting antimicrobial stewardship.

## Methods

This mixed methods convergent parallel study reports qualitative content in compliance with the Standards for Reporting Qualitative Research (SRQR)^36^, and an internet survey in compliance with Checklist for Reporting Results of Internet E-Surveys (CHERRIES) criteria^37^.

### Mixed methods approach and research paradigm

A mixed methods approach using quantitative and qualitative data from both an online survey and one-to-one semi-structured interviews was employed to explore healthcare professionals’ experiences. This study adopts an interpretivist approach, which emphasises understanding participants’ subjective experiences and perceptions to inform inductive thematic analysis of qualitative data, enabling future iterative app development. Usability frameworks employed in the study included the Standardized User Experience Percentile Rank Questionnaire (SUPR-Q)^38^, the Questionnaire for User Interaction Satisfaction (QUIS)^39^, and the System Usability Scale (SUS)^40^. These frameworks provided structured evaluation of user interface design, navigation, content clarity, and decision support relevance. The online survey was implemented using Qualtrics software. It also provided a contextual analysis of CDSS apps within the domains of value-based healthcare^41^ and antimicrobial resistance (AMR) mitigation. Each of these frameworks offered distinct strengths that were leveraged to provide a comprehensive evaluation.

### Researcher characteristics and Reflexivity

The research team comprised doctors, pharmacists, and mixed methods researchers. Reflexive practices, including ongoing team discussions every two weeks, were employed to minimise bias and acknowledge how researchers’ professional backgrounds may influence data interpretation.

### Context

Eolas Medical is a clinical decision support system (CDSS) app used in this context for antimicrobial prescription decisions. The study was conducted in a large London teaching hospital trust comprising three large and two smaller hospitals. In July 2024, the trust transitioned from using MicroGuide to Eolas Medical to deliver antimicrobial guidelines. The decision to adopt Eolas was taken at the organisational level, driven by a desire to consolidate multiple departmental guidelines onto a single CDSS app with broader NHS uptake. However, due to a commercial acquisition, this change was not preceded by formal piloting or end-user consultation, and limited onboarding support was provided. These factors contributed to resistance from some users, particularly in comparison to the previously used MicroGuide app, which had a simpler navigation structure. The CDSS app does not interact with or store patient data, nor does it require system access or integration. This context provided insights into real-world challenges and needs specific to antimicrobial prescribing. Eolas Medical provided historical data to demonstrate the CDSS app’s usage at the London teaching hospital over recent months (Table 2). While Eolas Medical is currently used by over 80% of NHS hospital trusts in England, its depth of implementation and user engagement varies significantly across organisations. At the study site, Eolas is primarily used to access antimicrobial guidelines, although other departments have adopted it for additional protocols.

**Table 2:** Eolas Usage Data from July 2024 (introduction) to January 2025

Month	Total Monthly Sessions	Unique Monthly Users	Total Sessions
Jul-24	143	9	31423
Aug-24	332	10	66896
Sep-24	1182	243	126464
Oct-24	5567	644	219509
Nov-24	5667	671	207824
Dec-24	5139	622	221870
Jan-25	5081	634	229380

### Sampling strategy

Participants of the survey were users of the Eolas Medical app, with email invitations sent to all app users at the hospital organisation. Purposive sampling was used to recruit healthcare professionals for the interviews, aiming for diverse representation of roles (e.g., doctors, pharmacists, prescribing nurse practitioners). Sampling sufficiency was determined by the diversity of perspectives and thematic data saturation once now new themes were being raised at interview. Historical usage metrics from Eolas Medical during September 2024 to January 2025 were also collected. Virtual interviews with prescribers provided an opportunity to understand the app’s usability, barriers faced by users, and themes related to trust, using a pre-piloted topic guide, as outlined in Table 3. Recordings were transcribed for analysis, ensuring data integrity. Participants’ roles, experience, and familiarity with microbiology guidelines were documented to contextualise findings.

**Table 3:** Interview Questions (Using SUPR-Q and QUIS Frameworks)

These interview questions were designed to facilitate a deeper exploration of the usability, accessibility, and relevance of the Eolas Medical app, informed by SUPR-Q and QUIS frameworks. The aim is to elicit subjective feedback and actionable insights for improvements.
1. App Usage Motivation: “What are your primary motivations for using the Eolas app in your daily workflow? Are there any specific tasks that drive its use?”
2. Accessibility in Urgent Scenarios: “How accessible do you find the antimicrobial prescribing guidelines in situations requiring urgent decisions?”
3. Guideline Relevance: “How relevant and up-to-date do you feel the antimicrobial guidelines on the app are compared to other resources?”
4. Impact on Workflow: “In what ways has the Eolas app impacted your workflow, especially when it comes to prescribing antimicrobials?”
5. Ease of Navigation: “How easy is it for you to navigate through the Eolas app? Are there any areas you find confusing or cumbersome?”
6. Clarity of Information: “Do you find the information on antimicrobial prescribing clear and easily understandable? Are there areas where the clarity could be improved?”
7. Training and Support Needs: “Do you feel adequately supported in terms of training for the app? What additional resources or support would make a difference?”
8. Trust and Reliability: “To what extent do you trust the information presented in the Eolas app for prescribing antimicrobials?”
9. Comparisons with Other Tools: “How does the Eolas app compare with other clinical support tools you use for antimicrobial guidance?”
10. Design Appeal: “What do you think of the app’s design and appearance? Do you feel it adds to or detracts from usability?”
11. Task Disruption vs. Support: “How often do you find the Eolas app either disruptive or supportive during a patient interaction (e.g. penicillin allergy)?”
12. Time Efficiency: “Do you feel the Eolas app helps save time when accessing antimicrobial guidelines, or do you find it slows you down?”
13. Customisability and Control: “How much control do you feel you have in customizing or filtering the guidelines to fit specific patient needs?”
14. User Satisfaction: “Overall, how satisfied are you with using the Eolas app for antimicrobial prescribing? Are there specific areas where it excels or falls short?”
15. Error Prevention: “Do you feel that using the Eolas app helps prevent errors in antimicrobial prescribing? Can you give an example?”
16. Workload Impact: “How does the Eolas app impact your cognitive workload during prescribing? Does it simplify or complicate your task?”
17. Feature Usefulness: “Which features do you find most useful, and are there any you consider unnecessary or confusing?”
18. Integration into Other Systems: “How beneficial do you think it would be for the Eolas app to integrate directly into other systems like Cerner?”
19. Future Improvements: “What changes would you recommend to improve the Eolas app’s usability for antimicrobial prescribing?”
20. Final Thoughts on Accessibility: “Do you feel the antimicrobial guidelines are accessible to all users regardless of their technical expertise or clinical experience?”

Inclusion criteria: healthcare professionals employed at the London teaching hospital organisation; regular users of antimicrobial guidelines in clinical decision-making through use of the Eolas Medical app.

Exclusion criteria: non-healthcare staff; healthcare professionals not involved in antimicrobial-related decision-making.

### Development and pre-testing

The survey was developed based on established usability evaluation frameworks such as SUPR-Q, QUIS, and SUS, and administered using Qualtrics. Pre-testing included piloting the survey with ten clinical research fellows and testing the survey’s technical functionality before full distribution.

### Survey administration

The survey was administered online via Qualtrics. Participants accessed it voluntarily through email invitations. The survey was available from December 2024 to January 2025. Completeness checks were built into the survey using Qualtrics’ validation features, ensuring mandatory fields were completed before submission.

### Preventing multiple entries

Qualtrics employed cookies and IP address checks to minimize duplicate entries. Users could not re-access the survey once submitted, reducing the risk of multiple entries. Additionally, data were reviewed for duplicate responses based on IP addresses and timestamps.

### Data processing

Data from interviews were transcribed verbatim using Microsoft® Teams Video Conferencing software. Anonymisation was conducted during transcription, and data were managed using secure, password-protected software. Qualitative data were coded and organised into themes using inductive reflective thematic analysis. The mixed methods analysis included surveys and prescriber interview video calls, where participants provided experiential input on the usability, applicability, and comprehensiveness of the guidelines. This enabled data triangulation by separate analysis and then discussion together. The interpretivist paradigm guided analysis, emphasising participants’ perspectives.

### Techniques to enhance trustworthiness

Credibility was ensured through triangulation of data sources (interviews, surveys, usage metrics), whilst clinician involvement also enhanced the reliability of findings.

### Ethical issues pertaining to human subjects

Service evaluation approval (Number 1090) was obtained from the NHS trust concerned. Participants provided written informed consent for both the survey and the interviews and were informed of their right to withdraw at any time without repercussions. Data security measures included anonymisation and storage on password-protected systems compliant with NHS data protection requirements.

## Supplementary information

Supplementary Information

## Data Availability

The data that support the findings of this study are available from the corresponding author upon reasonable request. Detailed usage data and the survey framework are provided in the supplementary materials.
