# Supplementary material for: A mixed methods evaluation of an antimicrobial prescribing clinical decision support system app
Source: NPJ Antimicrob Resist. 2025 Aug 18;3:71. doi: 10.1038/s44259-025-00146-8 (PMC12361472; doi:10.1038/s44259-025-00146-8)
Supplement: Supplementary file 1 — Supplementary Information [file 44259_2025_146_MOESM1_ESM.docx]

Supplementary Table 1: Interview and Survey Thematic Analysis

| **Theme** | **Sub-Themes** | **Representative Quotes** | **Data Source** |
| --- | --- | --- | --- |
| Primary Use | Antimicrobial Guidance | ‘The most important use of Eolas is prescribing antibiotics.’ | Interview |
| Primary Use | Frequently Used Features | ‘I use the Eolas app to ensure quick access to national guidelines and Trust policies on antibiotics.’ | Interview |
| Navigation | Ease of Access | ‘You have to be in the right place... It wasn’t intuitive in sending you to the local guidelines.’ | Interview |
| Navigation | Efficiency in Decision-Making | ‘It should be a bit more seamless. There should be less... it cannot be that many clicks till I get Microguide.’ | Interview |
| Navigation | Emergency Navigation Efficiency | ‘There are some areas... it just leads us to go through the other information that we don't need for the emergency situation.’ | Interview |
| Usability | Clutter and Redundancy | ‘You don’t need a million BNF [British National Formulary] links on Eolas.’ | Interview |
| Usability | Cognitive Load Reduction | ‘What I need is straightforward... I didn’t find Eolas intuitive or straightforward.’ | Interview |
| Usability | Transition Challenges | ‘I used to use Microguide all the time... then I couldn’t find the guideline for a while.’ | Interview |
| Design Appeal | Preference for Simplicity | ‘I liked Microguide because it gave you antimicrobial guidelines... There were no options for you to go wrong.’ | Interview |
| Training | Need for Intuitiveness | ‘Apps have to be incredibly intuitive... they shouldn’t need training.’ | Interview |
| Training | Onboarding Support | ‘The hardest part for me was linking the app with the [hospital name] Trust guidelines when I started.’ | Interview |
| Training | Awareness & Promotion | ‘I haven’t seen much promotion of the app within the trust.’ | Interview |
| Integration | System Compatibility | ‘Don’t integrate Eolas directly into systems like Cerner...’ | Interview |
| Integration | Integration with Clinical Systems | ‘It would be helpful if it was integrated directly in systems like Cerner.’ | Interview |
| Integration | External Database Integration | ‘If we had more compatibility... it would be really, really helpful.’ | Interview |
| Patient Safety | Decision Support Features | ‘Having the information side by side... reassures you and helps you.’ | Interview |
| Patient Safety | Protection Against Errors | ‘100%.’ (When asked if the app prevents errors) | Interview |
| Patient Safety | Potential Risks | ‘There’s still an overreliance on certain antibiotics leading to allergic reactions.’ | Interview |
| Customisation | User Preferences | ‘Make it a bit easier to access for every doctor to the first click on it... intuitive design is key.’ | Interview |
| Customisation | Personalised Guidelines | ‘The antimicrobial guidance just needs to be a big chunk of the main menu.’ | Interview |
| Feature Requests | Drug Interaction Features | ‘Maybe having drug interaction checks like in the BNF [British National Formulary] app would be useful.’ | Interview |
| Feature Requests | Integrated Calculators | ‘Having drug calculators integrated into the hospital guidelines section would be useful.’ | Interview |
| Feature Requests | Real-Time Prompts | ‘It would be good if, for example, it prompted you that this is not what the guidelines suggest.’ | Interview |
| Feature Requests | Intelligent Search | ‘It would be nice if you wrote ‘Fournier’s gangrene’ and it immediately came out.’ | Interview |
| Feature Requests | Surgical-Specific Guidelines | ‘Surgical prophylaxis... it needs to be easy.’ | Interview |
| Usage Patterns | Morning Use (8am-11am) | ‘At start of theatre list to confirm correct surgical prophylaxis to administer.’ | Survey |
| Usage Patterns | Midday Use (11am-2pm) | ‘Start of afternoon surgical list.’ | Survey |
| Usage Patterns | Afternoon Use (2pm-6pm) | ‘If called out of hours for advice to check our indications.’ | Survey |
| Usage Patterns | Evening Use (6pm-midnight) | ‘Most likely time I be on a late shift and busy either in resus or rapid assessment and treatment.’ | Survey |
| Survey Insights | Ease of Navigation | ‘Navigation is clunky - it’s essentially a word document, not an app, so navigation is either a jump from contents or scrolling.’ | Survey |
| Survey Insights | Efficiency in Decision-Making | ‘Microguide was simpler, I could reach decisions faster.’ | Survey |
| Survey Insights | Training Needs | ‘Would have been beneficial to get a quick induction on how to access the guide on the app, found it slightly confusing initially.’ | Survey |
| Survey Insights | Feature Suggestions | ‘Advanced improved search that takes you straight to the guideline.’ | Survey |
| Survey Insights | Frequently Used Features | ‘Local hospital guidelines, antimicrobial guidelines, surgical antimicrobial prophylaxis.’ | Survey |
| Survey Insights | Redundant Features | ‘NICE and other information as this can be accessed separately and may cause too much information.’ | Survey |

Supplementary Table 2: Eolas Medical App Survey to Prescribers

| Q1 Antimicrobial prescribing guidelines play a crucial role in managing infections in healthcare settings. Accessing antimicrobial guidelines on smart phone applications is a way of ensuring guidance is available at the point of prescribing. This project will explore the current utility and adoption of antimicrobial prescribing guidelines as presented via the Eolas Medical app at [hospital name] gathering structured feedback from healthcare professionals. This work has been approved as a service evaluation at [hospital name] (#1090). Do you confirm your written consent to your responses being included in the service evaluation? If you decide to take part, you are free to withdraw at any time without giving a reason. All information collected during the service evaluation will be kept strictly confidential.   - Yes (1) - No (2)   Q2 Are you a prescriber?   - Yes (1) - No (2)   Q3 Please indicate your role at [hospital name]::   - Foundation Doctor/SHO (1) - Core Trainee Doctor (2) - SpR (3) - Consultant (4) - Pharmacist (5) - Advanced Nurse Practitioner (6) - Infection Services (7) - Other; please use text box (8) __________________________________________________   Q4 How frequently do you use the Eolas Medical app to access antimicrobial prescribing guidelines?   - Daily (1) - Weekly (2) - Monthly (3) - Less Often Than Monthly (4) - Never (5)   Q5 When are you most likely to use the Eolas app for accessing antimicrobial guidelines and why?   - 8am-11am (1) __________________________________________________ - 11am-4pm (2) __________________________________________________ - 5pm-8pm (3) __________________________________________________ - 8pm-1am (4) __________________________________________________ - 1am-8am (5) __________________________________________________ - All of the above (6) __________________________________________________   Q6 How easy is it for you to navigate the antimicrobial prescribing guidelines on the Eolas app?   - 1 (Extremely Difficult) (1) - 2 (Difficult) (2) - 3 (Neither Easy Nor Difficult) (3) - 4 (Easy) (4) - 5 (Extremely Easy) (5) - Not Applicable (6)   Q7 To what extent do you agree with the statement that 'the Eolas app provides relevant information for prescribing antimicrobials'?   - 1 (Strongly Disagree) (1) - 2 (Disagree) (2) - 3 (Neither Agree Nor Disagree) (3) - 4 (Agree) (4) - 5 (Strongly Agree) (5) - Not Applicable (6)   Q8 To what extent do you agree with the statement that 'the Eolas app provides information in an efficient manner for prescribing antimicrobials'?   - 1 (Strongly Disagree) (1) - 2 (Disagree) (2) - 3 (Neither Agree Nor Disagree) (3) - 4 (Agree) (4) - 5 (Strongly Agree) (5) - Not Applicable (6)   Q9 To what extent do you agree with the statement that 'the Eolas app’s design meets your expectations for a clinical decision tool'?   - 1 (Strongly Disagree) (1) - 2 (Disagree) (2) - 3 (Neither Agree Nor Disagree) (3) - 4 (Agree) (4) - 5 (Strongly Agree) (5) - Not Applicable (6)   Q10 How clearly are the antimicrobial prescribing guidelines presented on the app?   - 1 (Extremely Unclear) (1) - 2 (Somewhat Unclear) (2) - 3 (Neither Clear Nor Unclear) (3) - 4 (Somewhat Clear) (4) - 5 (Extremely Clear) (5) - Not Applicable (6)   Q11 How well does the Eolas app support you in making antimicrobial prescribing decisions?   - 1 (Not Well At All) (1) - 2 (Slightly well) (2) - 3 (Moderately Well) (3) - 4 (Very Well) (4) - 5 (Extremely Well) (5) - Not Applicable (6)   Q12 How relevant are the antimicrobial prescribing guidelines on the Eolas app to your daily clinical practice?   - 1 (Not Relevant At All) (1) - 2 (Slightly Relevant) (2) - 3 (Moderately Relevant) (3) - 4 (Very Relevant) (4) - 5 (Extremely Relevant) (5) - Not Applicable (6)   Q13 How easy is it to complete specific tasks on the Eolas app? Why did you choose this option? Please consider giving multiple answers with text explanation (e.g., accessing dosage or allergy information).   - 1 (Extremely Difficult) (1) __________________________________________________ - 2 (Difficult) (2) __________________________________________________ - 3 (Neither Easy Nor Difficult) (3) __________________________________________________ - 4 (Easy) (4) __________________________________________________ - 5 (Extremely Easy) (5) __________________________________________________ - Not Applicable (6) __________________________________________________   14 Which additional training or support would improve your experience of using the Eolas app? Please explain your answers and consider giving more than one.  ________________________________________________________________  Q15 How efficiently can you achieve a prescribing decision with Eolas compared to decisions with other tools? Please give examples of better or worse experiences, using the comment boxes next to each answer.   - 1 (Extremely Inefficient - just as slow or slower than without any support) (4) __________________________________________________ - 2 (Somewhat Inefficient - slower than reading an intranet guidelines document) (5) __________________________________________________ - 3 (Neither Efficient Nor Inefficient - faster than reading an intranet guidelines document but slower than other clinical decision support apps) (6) __________________________________________________ - 4 (Somewhat Efficient - just as fast as other clinical decision support apps) (7) __________________________________________________ - 5 (Extremely Efficient - faster than any other clinical decision support app) (8) __________________________________________________ - Not Applicable (9) __________________________________________________   Q16 To what extent do you agree with the statement that 'the Eolas app always provides the precise antimicrobial prescribing information you need'?   - 1 (Strongly Disagree) (1) - 2 (Disagree) (2) - 3 (Neither Agree Nor Disagree) (3) - 4 (Agree) (4) - 5 (Strongly Agree) (5) - Not Applicable (6)   Q17 Which features of the antimicrobial prescribing Eolas app do you use most frequently?  ________________________________________________________________  Q18 Which features of the antimicrobial prescribing Eolas app do you find redundant or less helpful?  ________________________________________________________________  Q19 What one feature would you change or add to improve the app’s usability for antimicrobial prescribing?  ________________________________________________________________  Q20 To what extent do you agree with the statement that 'the value of the Eolas app would increase from being embedded in the electronic patient health record'?   - 1 (Strongly Disagree) (1) - 2 (Disagree) (2) - 3 (Neither Agree or Disagree) (3) - 4 (Agree) (4) - 5 (Strongly Agree) (5) - Not Applicable (6)   Q21 Please can you leave your name, role and email if you consent to be contacted to be invited to an interview to elaborate on your experience using the Eolas app to prescribe antimicrobials?   - Name (1) __________________________________________________ - Role (2) __________________________________________________ - Email (3) __________________________________________________ |
| --- |
